# The connotation of “old”: evidence of an expanded present from movies

**DOI:** 10.1007/s00426-025-02183-4

**Published:** 2025-11-28

**Authors:** Dirk Wentura, Patrick Schuck

**Affiliations:** Saarland University, Saarbrücken, Germany

## Abstract

We explore the concept of an *expanded present* by examining how individuals perceive the “age” of an event, product, or artefact in relation to their own age. We hypothesized that a certain age acts as a kind of tipping point for an “old” versus “new/recent/current” connotation. Events occurring before that age connote a halo of “oldness,” whereas events occurring after that age connote a halo of “recency.” We used movies as a case study and employed a response priming design, presenting movie posters from different decades (1960s to 2010s) as briefly presented primes, preceding target movie posters from the 1960s and 2010s, which had to be categorized as “old” or “new.” Participants were from four age cohorts (born in the 1960s, 1970s, 1980s, and 1990s). Results supported the hypothesis, showing that individuals tend to spontaneously categorize movies released in their adolescence or later as “new/current” and earlier movies as “old.”

**Supplementary Information:**

The online version contains supplementary material available at 10.1007/s00426-025-02183-4.

## The connotation of “old”: Evidence of an expanded present from movies

Older readers (like the first author, born in 1961) will be familiar with the following irritating phenomenon: When engaged in a conversation with someone much younger (e.g., a 25-year-old), they might refer to a particular event (e.g., 9/11), movie (e.g., *When Harry Met Sally*), or political era (e.g., the Clinton years) that took place long ago as if it took place in the near past. But to their conversation partner, the event will indeed feel very far away in time or might even feel as though it came from a “different age.” Often only analogies will help make the speaker fully aware of the discrepancy: “Mentioning 9/11 is to your conversation partner (the 25-year-old) what mentioning the JFK assassination was to you when you were 25.” In other words, to the 25-year-old, the event is wrapped in a halo of “old,” something that took place “before my time.”

The phenomenon does not seem to be related to personal knowledge about the issue. For example, both the JFK assassination and 9/11 were still quite prominent in the media even decades after the respective event. Thus, even very young people had full knowledge of the event, yet they still had the feeling that the event was “situated in a different age.” It also does not seem to be a matter of experience: Movies, pop songs, or novels are accessible for decades and are often well-received by both older and younger people. However, reception is accompanied by a subtle connotation of whether it is a more-or-less “recent” product or something from the “past.” For example, compare a 50-year-old senior researcher who is writing a manuscript with a 25-year-old graduate student who is writing a thesis. If they both wish to cite the same article from 2011, the senior researcher might have to suppress the urge to refer to it as “recent research,” while the graduate student will naturally refer to it as “early research.”

Our tentative term for this phenomenon is the *expanded present*, denoting a nonlinearity of our consciousness of things past with regard to their occurrence. The working hypothesis is that a certain age is a kind of tipping point for an “old” versus “new/recent/present day” connotation. Events that took place before this tipping point or products that emerged before it are surrounded by a halo of “oldness”—or are “not from my time”— whereas events/products occurring afterwards have a halo of “recency.” The tipping point is assumed to occur in young adulthood. This is initially just an intuitive assumption, but it is supported by the plausible idea that it requires individuals with fairly elaborate self-concepts (i.e., adolescents) who locate themselves in a specific historical period.

With the present study, we attempted a first-time exemplification of this phenomenon by referring to movies. Of course, a 63-year-old movie fan (such as the first author) *knows* that *When Harry Met Sally* is quite an old movie (it is from 1989). But how does he *feel* about this movie with regard to its age? Is it still a movie that feels as though it is part of an expanded present? So is this film, which is over 30 years old, still a “new” film for him in a sense, whereas *An Affair to Remember* (a Cary Grant movie from 1957) is an “old” film and was already old even when he first saw it in the late 1970 s (i.e., 20 years after it was released)?

How should we attempt to approach this phenomenon? Obviously, a direct question “Is this an old or a new film?” is not appropriate. Apart from needing to ask “What time span marks the difference between old and new?” the participants would likely base their answer mainly on their knowledge (which, e.g., indicates that *When Harry Met Sally* is quite an old movie for everyone). Here, we applied an indirect way to test our hypothesis: Participants were asked to quickly categorize unique cases of films as “old” or “new” (i.e., movies from the 1960 s vs. movies from the 2010 s). Target presentation was preceded by briefly presented movie posters, drawn from all decades between the 1960s and the 2010s. Spontaneously and involuntarily categorizing the primes as “old” or “new” in the context of this task was expected to facilitate or hamper participants’ responses to the targets, depending on the congruence or incongruence of the categorization with the target-related response. Most importantly, the hypothesis of the expanded present would suggest that it should depend on the participant’s age whether, for example, a movie from the 1980 s triggers an involuntary and spontaneous categorization as “new” or “old.”

In general, the method—a response priming design—is well-established and well-founded (although completely new for the test of the present hypothesis). The most well-known version of response priming is the evaluative priming paradigm (Fazio et al., 1986; Wentura & Degner, 2010). In evaluative priming, participants quickly categorize target stimuli that have a clear affective connotation as positive or negative. Targets are preceded by prime stimuli, which are completely task-irrelevant. If primes have a clear affective connotation as well, a congruence effect is typically found in response times: If the prime valence fits the response needed for the target (e.g., if both prime and target are positive; congruent condition), responses are faster than if the prime valence fits the counter category (e.g., if the prime is positive but the target is negative; incongruent condition). Moreover, this tool is also used to infer the connotation of primes: For example, if White participants are primed with Black and White faces, a priming effect can be calculated on the basis of a prejudice hypothesis if “Black faces/negative” and “White faces/positive” are taken as congruent and “Black faces/positive” and “White faces/negative” are taken as incongruent. If the mean priming score is positive, it can be taken as evidence that the faces involuntarily elicited the hypothesized affective connotations (Fazio et al., 1995; Degner & Wentura, 2011; Gurbuz et al., 2024; Paulus & Wentura, 2018).

**Fig. 1:**
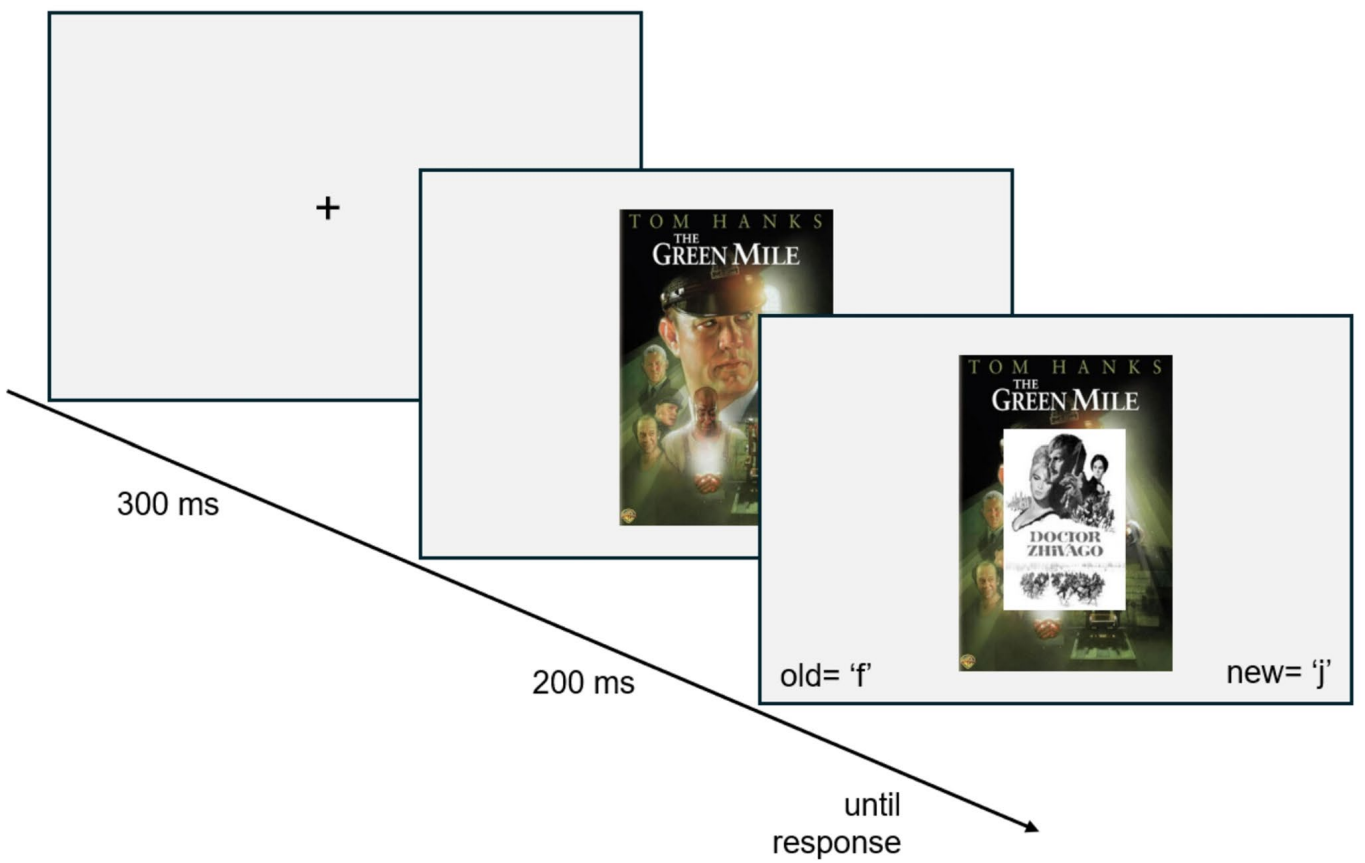
Trial Sequence of the Response Priming Procedure. Participant’s task was to categorize small black and white target stimuli as either “old” or “young”. Targets were either from the 1960 s (as Doctor Zhivago, 1965; “old”) or from the 2010 s, thus unequivocally old or young. To-be-ignored colored prime stimuli were from all decades from the 1960 s to the 2010s. The example Green Mile is from 1999. For participants of what age, does this prime trigger a spontaneous “old” (or “new”) categorization, thereby facilitating (or interfering with) the response to Doctor Zhivago? (Proportions are not true to scale; Copyright for the movie posters Warner Bros. Entertainment Inc.)

Here, we hypothesized that, in the context of an old/new categorization of target movies, the prime stimuli would involuntarily evoke a connotation of being old or new as well (see Fig. 1). Because we assumed that this connotation would depend on participants’ age, we expected a pattern of cohort-specific priming effects over the span of the priming decades. We recruited participants from four age cohorts (i.e., individuals born in the 1960 s, 1970 s, 1980 s, or 1990 s). Primes (i.e., movie posters) were taken from six decades (the 1960 s, 1970 s, 1980 s, 1990 s, 2000 s, and 2010 s, hereafter abbreviated as 60 s, 70 s, 80 s, 90 s, 00 s, 10 s). In our preregistration, as a first rough approximation of the “tipping point” (see above), the prime movies that we expected to be considered “new/recent” were the ones released two decades (or more) after the participants’ decade. That is, for participants born in the 60 s (70s, 80, 90 s), all movies from the 80 s (90s, 00 s, 10 s) and beyond were expected to be classified as “new/recent.”

## Method

### Research transparency statement

We report how we determined our sample size, any data exclusions, all manipulations, and all measures in this study. Raw data, analysis scripts, and materials can be found at https://osf.io/uwrzv.

We preregistered our experiment on aspredicted.org (https://aspredicted.org/XX6_X47).

### Participants

We proceeded from the assumption of an effect size of *d*_*Z*_ = 0.5 (i.e., medium; Cohen, 1988) for priming effects. To achieve a power of 1-β = 0.95 (α = 0.05, two-tailed) for each age group, we recruited 54 participants per cohort (G*Power; Faul et al., 2007). Age cohorts were the 1960 s, 1970 s, 1980 s, and 1990s.

We recruited data online (in December 2021) via Prolific (www.prolific.co) by addressing movie fans in particular. Filter criteria were fluent in English and the use of a desktop computer. To control for the number of participants in each cohort, the experiment began separately for each cohort. The *N* = 216 participants were preselected from a larger sample of *N* = 298 participants on the basis of performance on a quiz to ensure some level of knowledge of film history (see *Procedure*). Participants received 2.5 £ for their participation.

In line with our preregistered criteria, we discarded data from 22 participants who had error rates above 20%. With regard to our second preregistered criterion (i.e., mean RTs that were three interquartile ranges above the third quartile with respect to the distribution of mean RTs; Tukey, 1977), no participants had to be excluded. An unanticipated problem was that five participants reported an age that did not match the cohort in which they were recruited. We decided to discard these data because it was not clear which piece of information was valid.^1^

Thus, our final sample had *N* = 189 participants (see Table 1); the smallest cohort had 44, which was still sufficient to detect effect sizes of d_Z_ = 0.5 with power = 0.90.

**Table 1:** Sample characteristics

					Age	
Cohort	*N*	Women	Men		Median	Range
60s	44	20	24		56	52–61
70s	49	22	26		45	42–51
80s	47	26	21		36	31–41
90s	49	35	13		27	22–31

Two participants did not disclose their gender.

### Design

We employed a 6 (prime decade: 60 s, 70 s, 80 s, 90 s, 00 s, 10 s) × 2 (target decade: 60 s vs. 10 s) × 4 (age cohort: 60 s, 70 s, 80 s, 90 s) design with prime and target decades as within-participants factors and cohort as a between-participants factor.

### Materials

Two (for the 60 s and 10s: four) movie posters were selected from the nominees and winners of the Best Picture Academy Awards (“Oscars”) of each year from 1960 to 2019, for a total of 120 primes and 40 targets. As a rule, the Oscar-winning movie and the most famous nominee (as indicated by the score and number of ratings on *Rotten Tomatoes*, https://www.rottentomatoes.com) were selected as primes. The same applies to the 60 s and 10 s, with the third and fourth most famous nominees being selected as targets. Criteria for not including a movie were: if sequels or a remake existed or if the movie was too unknown, as defined by the relative number of ratings on *Rotten Tomatoes*. Another 20 posters, selected from either the nominees or other movies with high popularity ratings in their respective decades, were selected as training primes. All movie posters were edited to remove any mention of release year or Oscar award/nomination. The size of the primes was set to 275 (width) × 407 (height) pixels, and the size of the targets was set to 181 × 269 pixels.

### Procedure

At the beginning of the session, participants were asked to place the screen at a distance of their outstretched arm (about 65–75 cm). After completing the consent form, they were presented with a screen with instructions. To adjust presentation parameters to the actual screen size, participants were asked to resize a credit card image (presented on the screen) to the size of a real credit card (or equivalent) by pressing buttons.

The participants were preselected for the main task on the basis of their score on a quiz concerning Oscar-winning movies, ranging from the 60 s to the 10s.^2^ There were four multiple-choice answers to each of the 10 questions, so that the expected value in cases of guessing was 2.5. We set a criterion of five correctly answered questions for participation in the priming part.

In Phase 1, all primes were presented in random order, and participants indicated for each movie whether they had (a) not heard of it, (b) heard of it, or (c) seen it before. This prime task served two purposes: to somewhat familiarize participants with the primes and to use the knowledge data for control analyses (see Supplement).

Then the main task was practiced, in which all 40 targets were presented for the first time. Participants were instructed to categorize the targets from the 60 s as “old” and those from the 10 s as “new” as quickly as possible. No primes were presented during the first 20 trials. Then, participants were informed that the black and white target stimuli were preceded by slightly larger and briefly presented color movie posters, which they should ignore; target categorization with primes was practiced for 10 trials. Finally, the main phase began with 10 warm-up trials, unbeknownst to participants. For all practice trials, feedback was given, that is, after correct categorizations (“Correct Answer!”) as well as for wrong answers (“Wrong Answer!”). Thus, as all targets were presented once before the main trials began, erroneous categorizations should be minimized.

Each trial (see Fig. 1) began by presenting a fixation cross in the center of the screen for 300 ms, followed by the centered prime; 200 ms after the onset of the prime, the (smaller) target was presented superimposed on the prime until the response was given (or until a time-out of 2 s). Reminders of the key assignment (old = “f” in the lower left corner; new = “j” in the lower right corner) were presented in parallel to the target. Feedback was given after errors (“Wrong Answer!“) and time-outs (“Too Slow!“). At the end of each trial, a blank screen was shown for 500 ms before the next trial began.

The main phase comprised 240 trials in two blocks. Each prime appeared once in each block, in one block paired with an old target, in the other block paired with a new target, in accordance with the following counterbalancing schema: For half of the participants, odd year primes were assigned to an old target in Block 1 and to a new target in Block 2, whereas even year primes received the opposite assignment. For the other half of the participants, this odd/even assignment was switched. A self-paced break was included between blocks.

## Results

Mean RTs of correct trials served as the dependent variable. The error rate was 6.9% (*SD* = 4.4%). Trials with RTs below 150 ms or greater than 1.5 interquartile ranges above the third quartile with respect to the individual distribution of RTs were discarded (Tukey, 1977; 3.9% of trials).

### Preregistered analyses

We calculated simple priming differences by subtracting the mean RT for new-target trials from the mean RT for old-target trials, separately for all conditions of the Age Cohort × Prime Decade design. Table 2 shows these priming differences, which are centered row-wise to remove main effects of target type.

The nonshaded areas in Table 2 denote priming differences that refer to prime decades from before birth up to the decade in which the participant reached an age between 10 and 19. The grey-shaded areas denote priming differences that refer to prime decades in which the participant reached the age of 20. This procedure provided a rough implementation of the old/new tipping point.

**Table 2:**
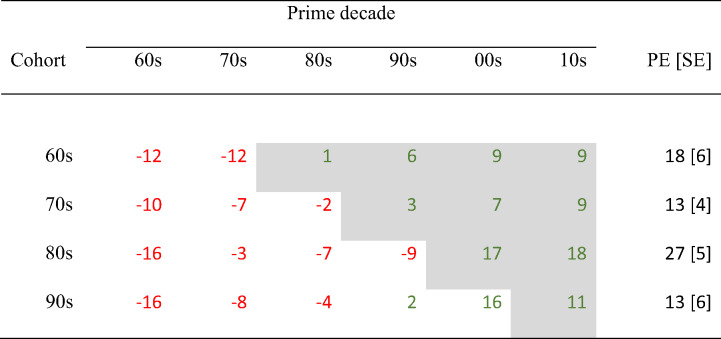
Simple priming differences as a function of cohort and prime decade and priming effects

*Note*. Simple priming differences (in ms) = mean RT_old-target trials_ - mean RT_new-target trials_. The differences were centered by subtracting the row mean. Positive values are printed in green; negative values are printed in red. The grey-shaded cells denote prime decades from before birth up to the age range 10–19 for the respective cohort. PE = priming effects = mean(grey-shaded cells) – mean(nonshaded cells); standard errors in parentheses

Note, the simple priming differences cannot be meaningfully tested on deviation from zero. Therefore, *priming effects* were calculated by subtracting the average simple priming differences referring to “old” decades for a cohort (nonshaded cells in Table 2) from average simple priming differences referring to “new” decades for a cohort (grey-shaded cells in Table 2; see the right-most column of Table 2). A one-way ANOVA with cohort as the between-participants factor and individual priming effects as the dependent variable yielded a significant constant effect (i.e., there was a significant overall priming effect), *F*(1, 185) = 39.98, *p* <.001, η_p_² = 0.178 (*d*_*Z*_ = 0.46), and—as expected—no effect of cohort, *F*(3, 185) = 1.36, *p* =.257, η_p_² = 0.022

**Fig. 2:**
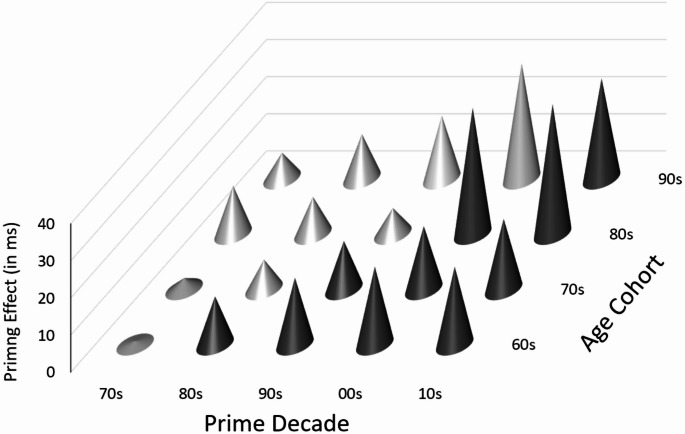
Priming Effects (Baseline 60 s Primes) for All Prime Decades (From the 70 s on) and Cohorts. Priming Effects were calculated by subtracting simple priming differences of each decade (beyond the 60 s) and each cohort from simple differences for the 60 s prime decade. We calculated simple priming differences by subtracting the mean RT for new-target trials from the mean RT for old-target trials. A positive value for Decade x in Cohort y means that, relative to a 60 s prime, the x primes relatively favored the “new” response. The light grey cones denote prime decades from before birth up to the age range 10-19 for the respective cohort, the dark grey cones denote prime decades thereafter

In an additional preregistered analysis, we calculated *priming effects* (not only simple differences) for all cells of the 4 (Cohort) × 5 (Prime decades: 70 s to 10 s) matrix by taking the simple differences for the 60 s prime decade as the baseline, which was subtracted from the simple differences of each remaining decade. A positive value for Decade *x* in Cohort *y* means that, relative to a 60 s prime, the *x* primes relatively favored the “new” response. Figure 2 shows these differences. As can easily be seen, *cohort-wise* the light-grey cones were comparably (i.e., within-cohort) low, whereas the dark-grey cones were comparably high. One marked deviation was immediately evident (and could already be anticipated from Table 2): The 90 s cohort showed a marked priming effect for the primes referring to movies from the first decade of the century (when participants were between 10 and 19 years old).

The decade-wise priming effects were somewhat noisy because they were based on only 40 trials each: 20 for the simple difference for the 60 s primes and 20 for the simple difference for the respective decade. Nevertheless, *t* values (M/SE) were between 1.5 and 4.4 (*Mdn* = 2.3) for all dark-grey cones in Fig. 2, but 0.1 to 2.2 (except 4.1 for the 00 s priming effect in the 90 s cohort; *Mdn* = 1.17) for the light-grey cones. To reduce the noise in a way that was unbiased with regard to the comparison of prime decades 70 s to 10 s, we tested the simple differences in these decades against the cohort-wise mean 60 s simple difference (i.e., against a constant value), with row-wise Bonferroni adjustment (i.e., *p* <.01, one-tailed, for a single test).

For the 60 s cohort, these tests yielded *t*(43) = 2.49 to 3.74, all *p*s < 0.01, for the four decades 80 s to 10 s, but *t* < 1 for the 70 s decade. For the 70 s cohort, these tests yielded *t*(48) = 2.48 to 3.56, all *p*s < 0.01, for the three decades 90 s to 10 s, but *t*(48) = 1.51, *p* =.069, and *t* < 1 for the 80 s and 70 s decades, respectively. For the 80 s cohort, these tests yielded *t*(46) = 6.61 and 7.30 (both *ps* < 0.001) for the two decades 00 s and 10 s, respectively, but *t*(46) = 1.41 (*p* =.082) and *t*(46) = 2.06 (*p* =.023) for the 80 s and 90 s, respectively. A significant result for the 70 s, *t*(46) = 3.56 (*p* <.001) went against our expectations. Thus, up to this point, only one out of 15 results did not match our expectations.

The 90 s cohort behaved somewhat differently. First, as already noted above, the primes for the 10 s *and* the 00 s decades showed a marked priming effect, *t*(48) = 5.05 and 6.65 (both *p*s < 0.001), respectively. Second, the *p* <.01 criterion was also met for the 80 s and 90 s decades, *t*(48) = 2.88 (*p* =.003) and *t*(48) = 4.37 (*p* <.001), respectively. Only the effect for the 70 s was not significant, *t*(48) = 1.47 (*p* =.074), as expected.

### Additional analyses

We added some analyses that were not preregistered. These analyses served two purposes. First, they were more fine-grained in that they took the actual age of participants and actual year of the primes into account. Second, there are some caveats that must be addressed.

For each participant, we calculated an age-dependent priming effect by (a) aggregating simple priming differences (i.e., mean RT_old−target trials_ - mean RT_new−target trials_) for movies from the early years, including the year in which the participant turned 15 (“preperiod”) and (b) aggregating simple differences for prime movie for all years from when participants were 16 or older (“postperiod”), and finally subtracting the preperiod aggregate from the postperiod aggregate to obtain a priming score. The mean priming score was *M* = 21 ms (*SE* = 3 ms), *t*(188) = 7.29, *p* <.001, *d*_*Z*_ = 0.53.

**Fig. 3:**
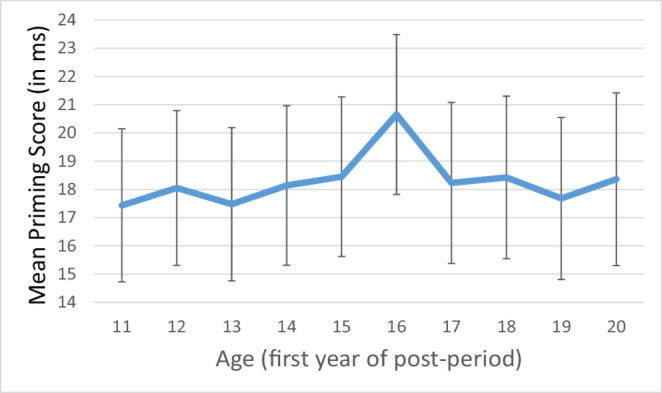
Individualized Priming Scores (in ms) for a Range of Cut-Off Years (First Year of the Postperiod +/−1 SE). For each participant, we calculated an age-dependent priming effect by (a) aggregating simple priming differences (i.e., mean RT_old−target trials_ - mean RT_new−target trials_) for movies from the early years, including the year in which the participant turned *x* (“preperiod”) and (b) aggregating simple differences for prime movie for all years from when participants were *x + 1* or older (“postperiod”), and finally subtracting the preperiod aggregate from the postperiod aggregate to obtain a priming score. The Figure shows the priming scores for cut-off values x from 11 to 20 years.

The cut-off value of 16 years was chosen intuitively. However, to support this decision, we analyzed cut-offs from 11 to 20 years, too. Figure 3 shows the mean priming scores for this range of cut-off values. As can be seen, the peak indeed occurred for the initially chosen cut-off. Thus, we proceeded with this priming score.

As said, there are some caveats that must be addressed. Of course, for each individual priming score, the very early years (i.e., “old” primes for everyone) entered into the preperiod value, and the very late years (i.e., “new” primes for everyone) entered into the postperiod value. Thus, if we calculated, for example, the priming score for all participants as if they were 43 years old, we would obtain a significant overall priming effect as well. So we had to ensure that the individual split—based on participants’ age—provided a particularly pronounced priming effect. We used two means to provide evidence for this effect.

**Fig. 4:**
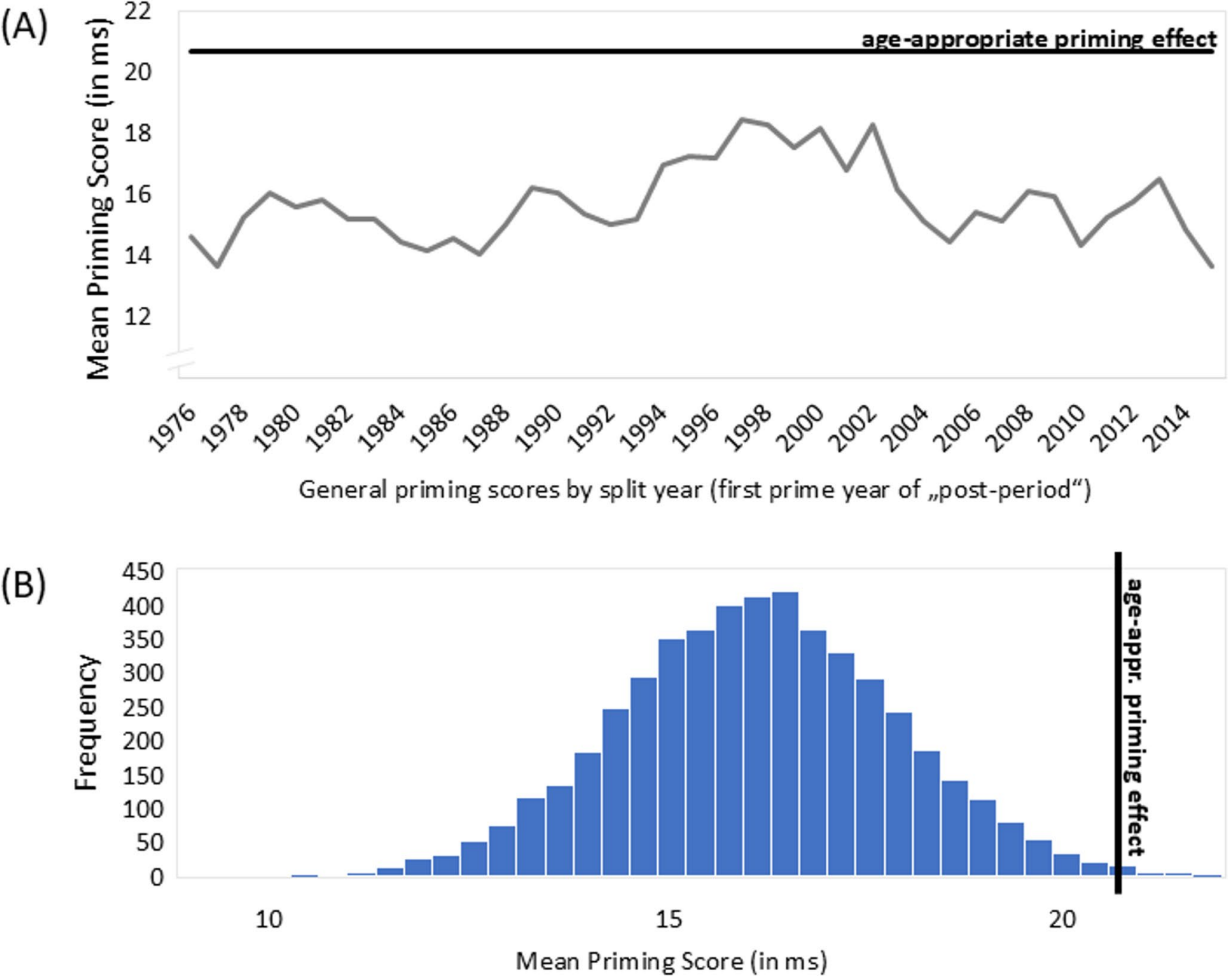
The Age-Appropriate Priming Score (Based on Participants’ Individual Age, cut-off 16 years; Black Lines) in Relation to (A) General Scores for All Participants as a Function of Split Year (Grey Line) and (B) the Distribution of Bootstrapped Mean Priming Scores

First, Fig. 4A shows the distribution of the mean priming scores, each calculated by a constant split between the preperiod and postperiod for all participants.

The varying grey line in Fig. 4A shows the means of these age-related priming scores (e.g., if the scores were calculated for all participants as if they were 61 years old, that is, with the prime years 1976 to 2019 as “new” ones, a priming score of almost 15 ms would be obtained). The straight black line indicates the mean individual priming score (see above). As can easily be seen, this score clearly exceeded the non-individualized ones.

Second, in a bootstrapping procedure with *N* = 5,000 runs, in each run, we randomly assigned one of the age-dependent priming scores to each participant. For example, in a given run, the score that—according to the hypothesis—fit best for a participant aged 61 might be randomly assigned to a participant aged 35, a participant aged 44, or both (i.e., sampling with replacement). We then determined the distribution of the average priming scores across the 5,000 runs (see Fig. 4B). As can be seen, the overall priming score that was based on the hypothetically best split (i.e., up to when a participant was 15 as preperiod primes) belongs to the 0.5% largest priming effects based on random assignment.

Finally, we ran some control analyses with regard to whether participants’ knowledge of the prime movies moderated the results. These analyses can be found in the Supplement. In a nutshell, we found no evidence that participants’ knowledge played a role.

## Discussion

What do individuals perceive as “old” and what do they perceive as “new/present day”? Here, we suggested the concept of an expanded present that encompasses all years from adolescence to the actual present. This concept is not about knowledge because individuals have quite a good estimation of the age of certain events or cultural products on the basis of reconstructive memory; rather, this concept is about a connotation. Events/products that occurred (or were first introduced) early in a person’s life or before they were born (subjectively “old” events/products) have a halo of “not from my time” and a connotation of being old-fashioned even if the event (e.g., 9/11) or product (a movie, a pop-song, a type of furniture, a technical device) is still omnipresent in media. People who were already in young adulthood when the event happened or the product was introduced will not feel any disruption or mismatch when they encounter it again (although it is of course clear to them that the product is factual quite old). To provide initial evidence for this phenomenon, we chose films as a content area for illustrating the basic idea of the expanded present. And we proposed a simple method for capturing this “felt old” versus “felt present” difference, namely, a version of response priming.

Results were largely in accordance with our hypothesis. The Age Cohort × Prime Decade matrix of simple priming differences showed a pattern that conformed to the basic hypothesis: If the movie primes that were released at least two decades after the participants’ birth decade were aggregated and hypothetically taken as “new,” whereas all older movies were hypothetically taken as “old,” a clear priming effect emerged in support of the hypothesis. With one exception, even the single priming effects of the Prime Decade × Cohort Matrix, generated by always considering the 60 s primes as “old” and each other decade hypothetically as “new” supported the hypothesis: Within a cohort, the priming effects for the younger decades were always the strongest. The only exception was the priming effect for films from the 00 s in the youngest cohort. (We will return to this issue below.)

### How does the concept of the expanded present relate to other concepts or theories??

The concept of expanded present evokes associations to some other concepts of older and younger psychology. We will briefly discuss some of these associations.

In very general and abstract terms, the concept of the extended present could be reminiscent of George Herbert Mead’s “Philosophy of the Present” (Mead, 1932). Mead – philosopher and social psychologist, known especially for the theory of symbolic interactionism – emphasizes that the present is not simply the single moment in time which is already passed in the next moment; just to contrary, psychologically the present is decisive in reconstructing the past and constructing the future. Somehow, past and future exist only in the present (see also Flaherty & Fine, 2001; Järvinen, 2004). Thus, the idea of an expanded present developed in this article aligns with Mead’s ideas. But of course, these are broad abstract ideas that do not directly lead to the hypotheses and tests of the present article. Nevertheless, two thoughts related to the present topic are triggered by Mead’s theory. First, if the past is a reconstruction within the present, we should be cautious not to overstate the point that there is a fixed time point in adolescence that marks the beginning of an expanded present. For example, it is conceivable that significant life changes cause a new division between the “old” and the “new.” Or, another example, with advancing age, there might be some drift of the category boundary, perhaps not at the same speed as advancing chronological age, but some. (But note, we have no indications for that.) Second, Mead addresses both past and future as (re-)constructed from the perspective of the present. Up to this point, we wrote only about the past as divided into the expanded present and the past beyond the expanded present. One can easily transfer this way of thinking to the future: An expanded present with respect to the future means to extrapolate the present into the future insofar constancy of life circumstances (i.e., residence, job, social relationships) is expected. However, it is beyond the present article to further elaborate on this suggestion.^3^

A close connection to our topic can be found in the field of autobiographical memory. Autobiographical memory concerns a person’s memory for their own life events, life experiences, and autobiographical facts (e.g., Janssen & Murre, 2008). It therefore encompasses aspects of both episodic memory (i.e., the retrieval of specific episodes) and semantic memory, insofar as it involves facts about the self. Two features link the concept of the expanded present with research on autobiographical memory.

First, autobiographical memory includes not only episodes but also semantic knowledge. Referring to considerations by Conway and Pleydell-Pearce (2000), Prebble et al. (2013) describe “semanticized” autobiographical knowledge, which abstracts from individual episodes to form a life story, life themes, or general events (e.g., my memory for “how it was to be on a certain annual conference during the post-doc years”). It is perhaps this “semanticized” autobiographical knowledge which is addressed by the concept of expanded present. In basic memory research, Tulving (1993) emphasized that episodic and semantic memory differ with regard to the type of conscious awareness that accompanies retrieval of episodes and knowledge, respectively. It is the *autonoetic consciousness* of remembering an episode as part of the own past that defines episodic memory. Retrieving simple facts from semantic memory misses this kind of self-awareness. “Semanticized” autobiographical knowledge, however, is still accompanied by *autonoetic consciousness.* The presence versus missing of autonoetic consciousness might then be a correlate of the “new/present” versus “old” distinction put forward in this article.^4^

Second, in an influential article, Conway (2005) elaborates on the connections between memory and the self, emphasizing the role of autobiographical memories and autobiographical knowledge in creating a coherent self-concept. Coherence, in this context, refers to the consistency and interconnectedness of self-beliefs – that is, the mental representations of the self. Habermas and Bluck (2000) discuss four types of coherence, including temporal coherence. Although they primarily refer to the temporal ordering of a “life story” (see also Bluck & Habermas, 2000), the concept of the expanded present can be readily situated here. While we can order events, life periods, and products of popular culture in a temporal sequence, they are also connected to each other and to the self-concept. This interconnectedness is mirrored in the sense of coherence present in our phenomenological consciousness. Such a sense of coherence regarding one’s own past may form the basis for what we term the “extended present.” Thus, there is no contradiction between knowing the age of something and the feeling that it remains part of one’s life.

The immediate experience, the contact of the event/product with one’s personal life seems to be the key. Take, for example, two dramatic events: 9/11 and the assassination of John F. Kennedy. For a majority of readers of this article, the first one is presumably part of their personal experience, whereas the latter is before their time. Thus, for these readers, 9/11 is an event with personal involvement due to the close and immediate connection of reports about the event with their personal life. Interestingly, it is now known that so-called *flashbulb memories* (i.e., one’s memory of the personal context in which one learned about an event such as 9/11) decline in accuracy over time just like other autobiographical memories, but ratings of vividness do not (Talarico & Rubin, 2003). In contrast, the assassination of John F. Kennedy remains distant, even if the reports on the assassination and its consequences were accurately and comprehensively received (so that one cannot assume a difference in knowledge compared to 9/11). The Kennedy case feels almost as distant as historical events from the first decades of 20th century.

Beyond autobiographical memory research, Trope and Liberman (2010; see also Trope et al., 2021) introduced the concept of *psychological distance*: “Psychological distance is a subjective experience that something is close or far away from the self, here, and now. Psychological distance is thus egocentric: Its reference point is the self, here and now, and the different ways in which an object might be removed from that point—in time, space, social distance, and hypotheticality—constitute different distance dimensions” (p. 440). In this regard, the psychological distance in time seems to be a nonlinear function of objective time. Events from before birth to adolescence are very distant, whereas later events are closer.

The concept of *psychological distance* helps to better classify the phenomenon of *expanded present* since psychological distance is the more general term. For example, one can see similarities and analogies to other forms of distance and nearness. Take, for example, as an analogy to the contrast ‘past within the expanded present versus past outside’ the contrast ‘travel destinations that I have already visited versus travel destinations that I want to see (but have not visited yet)’. The mental representation of the latter consists of imaginations of the destination and the stay there, fed by reports, pictures, and videos. These imaginations remain distant from the self because they lack direct experience. The former is based on one’s own experiences of what it is like to be there. This can sometimes be very sobering when one realizes that on the way from attraction A to attraction B, one has to pass through an industrial park and a garbage dump. But nevertheless the place is now part of the self.

### A start of expanded present in adolescence??

Habermas and Bluck (2000) place the development of the life story in adolescence, which aligns well with our observation that the expanded present seems to begin then. In this regard, it is worth recalling studies typically cited as evidence for the “reminiscence bump,” that is, “the effect that people recall more personal events from early adulthood than from childhood or adulthood” (Janssen et al., 2007, p. 755). These studies go beyond that. They share one common feature: participants’ emphasis on products, people, and events from their young adulthood. For example, Holbrook and Schindler (1989) found a preference for pop songs released during participants’ young adulthood. Janssen et al. (2012) asked participants “to name the five football players who they considered to be the best players of all time” (p. 168); participants tended to nominate players whose career midpoint occurred during their adolescence. Janssen et al. (2007) asked participants to name their three favorite books, movies, and records; again, nominations clustered around products first encountered in young adulthood. Finally, Schuman and Scott (1989, p. 359) asked participants “to report ‘the national or world events or changes over the past 50 years’ that seemed to them especially important,” with a similar tendency to select events from adolescence and young adulthood.

Some of these results may indeed be due to the increased memory accessibility of the nominated events, products, or individuals. However, in studies such as Holbrook and Schindler (1989) and Janssen et al. (2012), participants were provided with lists of pop songs or football players from which to select. Thus, the results here reflect not merely memory accessibility, but also an emphasis and weighting that accompany the onset of a process linking not only the loose ends of personal experiences within the narrow context of one’s own life environment, but also connecting the self with the public world.

How can the “reminiscence bump” be reconciled with the concept of the expanded present, given that the former is a ‘peak’ phenomenon (the significance of events or products in youth is more prominent than that of events or products before or after), whereas the latter is a ‘plateau’ phenomenon (starting in adolescence, events or products carry a sustained new/present connotation)? The peak of the reminiscence bump initially serves primarily as a dividing line between what came before and what was experienced during youth. While earlier events or products were processed in isolation (if they were registered at all), those from youth are processed with richer references to the self-concept. They are not only more memorable but also imbued with meaning that fosters a perceived temporal proximity, even much later. Although subsequent events or products may not attain the same level of significance, they are still richly encoded—encountering a highly differentiated self-concept—while the factual temporal order remains intact. Thus, if events or products from youth retain the connotation of the new/present due to their anchoring in that period, later events or products inherit the same connotation by virtue of their temporal classification in relation to that of events or products of adolescence.

### Limitations and outlook

The present study represents only a first step. It provides initial support for the basic ideas underlying our hypothesis but does not constitute a strong test. An interesting limitation emerges in the priming effect for films from the 2000 s in the youngest cohort, which stands out as a striking exception to the general pattern. One plausible explanation for this exception is that the availability of films has changed—particularly for the youngest cohort in our sample—due to the introduction of streaming platforms. This shift may have weakened the association between films and their release years, especially for films from the past two decades, which are likely among the most frequently streamed. While this exception highlights a limitation of our study, it also points to a potentially broader cultural transition with implications for the phenomenon of the expanded past—namely, the permanent availability of all cultural products as a result of digitization (see, e.g., Hoskins, 2017, for a discussion).

Our study focused only on one content area—movies—although the basic idea is a more general one. It encompasses all areas of culture. We already gave the examples of outstanding events (e.g., 9/11) or the subjective age of scientific publications for senior and junior researchers (see *Introduction*). Other examples include fashion, home furnishings, and consumer electronics. Of course, a video recorder, a cathode ray tube TV, or a mobile phone from the presmartphone era are outdated technologies for everyone, including older people; however, the immediate feelings generated from seeing such devices are certainly very different for 60- and 20-year-old observers. This difference might not make a big difference in communication between people from different generations because, at a propositional level, there is no disagreement: “9/11 was in 2001”; “*When Harry Met Sally* is quite an old film”; “Video recorders are outdated.” However, the connotations that were the focus of this article are possibly so different that one might be inclined to say: They—the 60-somethings and the 20-somethings—are living in different worlds.

## Supplementary Information

Below is the link to the electronic supplementary material.

Supplementary file 1 (DOCX 116KB)

## Data Availability

Raw data and materials can be found at [https://osf.io/uwrzv].

## Footnotes

1 Including these participants did not essentially change the results.

2
https://www.funtrivia.com/playquiz/quiz3320772604220.html.

3 As a footnote, it would be a false pursuit of theoretical “elegance” to extrapolate the length of the past-related expanded present into the future; the asymmetry between the immutability of the past and the unpredictability of the future alone prevents this from happening. Nevertheless, a 20-year-old’s ideas about life at 40 will be just as “distant” and unsupported by experience as his ideas about life at 20 in the year of his birth. On the other hand, a 60-year-old will have an image of himself as an 80-year-old consisting of an extrapolation of his life over the past years or decades. (Of course, this image is supposedly embellished on average because it does not take into account the frailties of old age.) Given this perspective, one of the challenges of growing old becomes more evident: From a certain point onward, the expanded present will encompass a time period exceeding the expected life expectancy. Then, death becomes even more threatening because it is almost “now.”

4 A further association that should be mentioned is “nostalgia”, which has become an increasingly popular research topic in psychology in the last decade (Becker & Trigg, 2025; Sedikides & Wildschut, 2023). According to the Oxford English Dictionary, Nostalgia means a “sentimental longing for or regretful memory of a period of the past” (Oxford University Press, 2024). In their seminal article, Wildschut et al. (2006, p. 976) are more specific in specifying “nostalgia refers to a personally experienced past.” In this regard, there may be some overlaps to our ideas. However, nostalgia is a feeling and research focuses on affective consequences of nostalgic experiences whereas the concept of “expanded present” relates more to phenomenal consciousness, that is, the qualitative differences between timed periods with and without “me-ness”.
