# Supplementary material for: The connotation of “old”: evidence of an expanded present from movies
Source: Psychol Res. 2025 Nov 28;89(6):182. doi: 10.1007/s00426-025-02183-4 (PMC12662928; doi:10.1007/s00426-025-02183-4)
Supplement: Supplementary file 1 — (DOCX 116KB) [file 426_2025_2183_MOESM1_ESM.docx]

**Supplement**

**to *The Connotation of “Old”: Evidence of an Expanded Present From Movies***

Outline

1. Figure S1 (see main text, p. 13, footnote)
2. Figure S2 (see main text, p. 14)
3. The role of knowledge (see main text, p. 14)

**Figure S1**

*Individualized Priming Scores (in ms) for a Range of Cut-Off Years (First Year of the Postperiod +/-1 SE)*

**Figure S2**

*The Distribution of Bootstrapped Mean Priming Scores*


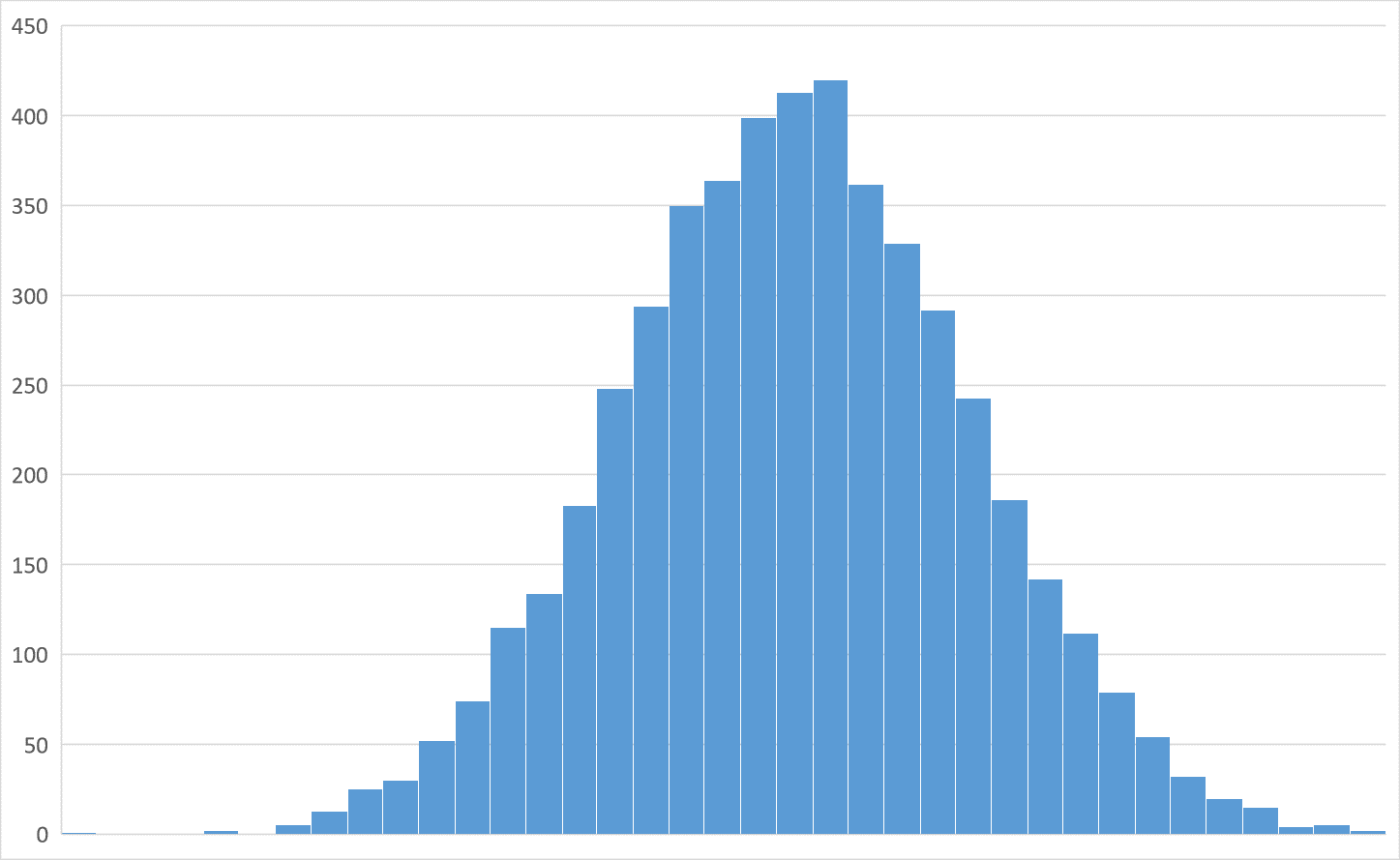


20

15

10

Mean Priming Score (in ms)

*Note*. The black line indicates the actual age-appropriate priming effect (see text for further explanation).

**The role of knowledge**

In the first phase of the experiment, we obtained knowledge data for the 120 primes, that is, participants reported whether the movie was “unknown” (1), “known, but not seen by them” (2), or whether they have seen it (3). In this Supplement, we provide some analyses, with regard to the question whether the results reported in the main text are moderated or compromised by variation in knowledge.

Table S1 shows descriptive data with regard to knowledge. As can be seen, there is indeed quite good knowledge of the prime movies. Not unexpectedly, however, there cohort differences especially for the early prime decades. Thus, it is worthwhile to explore the role of knowledge with regard to the priming effects.

*Table S1*. Proportion of known movies as a function of cohort and prime decade

|  |  | | | | | |  |  |
| --- | --- | --- | --- | --- | --- | --- | --- | --- |
|  | Prime Decade | | | | | |  |  |
| Cohort | 60s | 70s | 80s | 90s | 00s | 10s |  | Average |
|  |  |  |  |  |  |  |  |  |
| *Known^a^* | | | | |  |  |  |  |
| 60s | 0.80 | 0.84 | 0.93 | 0.89 | 0.77 | 0.63 |  | 0.81 |
| 70s | 0.71 | 0.70 | 0.79 | 0.92 | 0.83 | 0.65 |  | 0.77 |
| 80s | 0.61 | 0.62 | 0.64 | 0.85 | 0.88 | 0.73 |  | 0.72 |
| 90s | 0.52 | 0.48 | 0.47 | 0.73 | 0.71 | 0.75 |  | 0.61 |
|  |  |  |  |  |  |  |  |  |
| *Seen* | |  |  |  |  |  |  |  |
| 60s | 0.46 | 0.49 | 0.53 | 0.62 | 0.36 | 0.29 |  | 0.46 |
| 70s | 0.34 | 0.36 | 0.42 | 0.68 | 0.46 | 0.34 |  | 0.43 |
| 80s | 0.27 | 0.26 | 0.26 | 0.58 | 0.51 | 0.38 |  | 0.38 |
| 90s | 0.21 | 0.14 | 0.15 | 0.38 | 0.37 | 0.36 |  | 0.27 |

^a^ Ratings “known, but not seen it” and “seen”.

Does the mere knowledge of the prime movies predict priming? That is, does presentation of unknown movies bias towards an “old” (or else “new”) response whereas for known movies the opposite holds? We calculated two priming effects based on the knowledge rating. To obtain a first priming effect, we calculated simple priming differences by subtracting the mean reaction time of new-target trials from the mean reaction time of old-target trials, separately for “unknown” and “known” movies (collapsed over the rating categories “known, but not seen” and “seen”). Priming effects were calculated by subtracting the average simple priming differences referring to “unknown” movies from average simple priming differences referring to “known” movies. Column “PE_ks_” of Table S2 shows these effects. A positive value indicates that “known” prime movies bias towards “new” responses and/or “unknown” prime movies bias towards “old” responses. A one-factorial ANOVA with cohort as the between-participants factor and individual priming effects (PE_ks_) as the dependent variable yielded neither a significant constant effect (i.e., overall there is no significant priming) and no effect of cohort, both *F* < 1.

*Table S2*. Simple priming differences (in ms; centered row-wise) as a function of cohort and knowledge and priming effects (in ms; standard errors in brackets)

|  |  |  | | |  |  | | |
| --- | --- | --- | --- | --- | --- | --- | --- | --- |
|  |  | “unknown” (u) vs. “known + seen” (ks) | | |  | “unknown” (u) vs.  “seen” (s) | | |
|  |  |  |  |  |  |  |  |  |
| Cohort |  | u | ks | PE_ks_ |  | u | s | PE_s_ |
|  |  |  |  |  |  |  |  |  |
| 60s |  | -4 | 4 | 9 [11] |  | -3 | 3 | 7 [11] |
| 70s |  | 1 | -1 | -3 [6] |  | -2 | 2 | 4 [7] |
| 80s |  | 0 | 0 | -1 [8] |  | -2 | 2 | 3 [10] |
| 90s |  | -5 | 5 | 9 [7] |  | -5 | 5 | 10 [9] |
| Overall |  | -2 | 2 | 4 [4] |  | -3 | 3 | 6 [5] |

*Note.* Simple priming differences (SPD) are defined as mean RT_old-target trials_ - mean RT_new-target trials_; PE_ks_ = SPD_known+seen_ – SPD_unkown_; PE_s_ = SPD_seen_ – SPD_unkown_

We calculated a second priming effect (PE_s_) by contrasting “unknown” movies only with “seen” movies (disregarding “known, but not seen” primes). As can be seen from Table S2, this does not change much in comparison to PE_ks_. A one-factorial ANOVA with cohort as the between-participants factor and individual priming effects (PE_s_) as the dependent variable yielded neither a significant constant effect (i.e., overall there is no significant priming) and no effect of cohort, both *F* < 1.74.

Does knowledge modify the priming effects caused by prime age? To explore this issue, we conducted linear mixed models (lmm). We used the lmerTest package (Kuznetsova et al., 2016) which is based on lme4 (Bates et al., 2015) within the R environment for statistical computing (R-Core-Team, 2016) to run linear mixed models (LMMs). The package lmerTest delivers estimations of degrees of freedom (using Satterthwaite’s approximation) and accordingly *p*-values for the tests of the regression weights.

First, to rebuild our main analysis in the lmm format we regressed response times of trials (omitting error trials and trials with outlying values; see main text) on a variable coding individual prime age (P) with values +1 for primes that were released when the participant was 16 years old or older (i.e., hypothetical “new” primes) and -1 for primes that were released earlier (i.e., hypothetical “old” primes), on a variable coding target identity (T) with values +1 for targets of the 2010s years (i.e., the new targets) and -1 for targets of the 1960s years (i.e., the old targets), and the product term of the two coding variables. We specified the full model with random intercepts and random slopes for all predictor variables. Table S3 showed the results (header *Base Model*). As can seen, the interaction term P × T was significant. This result corresponds to our priming analysis with aggregated data.

The next analyses included the knowledge variable (K) with values +1 for primes that were known or even seen by participants and -1 for unknown primes. We ran analyses that included K and K × T additionally to the Base Model (i.e., P, T, and P × T) to see whether P × T succeeds in competition with K × T. The model including random slopes for all predictor terms did not converge. Therefore we removed random slopes for all terms involving the target variable. To see more clearly which changes are due to including additional predictors and which are due to removing random slopes for target terms, we first report the base model without random slopes for targets (header “Step 1” in Table S3) and then the model additionally including K and K × T (header “Step 2” in Table S3). As can be seen, the competition is clearly won by P × T; K × T does not essentially contribute to the prediction of response times.

In the next step, we checked whether knowledge *moderates* the priming effect (i.e., P × T). Therefore, we ran the full model, additionally including P × K and P × T × K. It can seen (header “Step 3” in Table S3) that the triple interaction was not significant. Finally, despite the non-significant triple interaction, we again conducted the base model, this time excluding trials that had primes unknown by the participant. The results are largely comparable to the base model comprising all trials (see Table S3; header “Base Model (without unknown primes)”).

*Table S3.* Results of the Linear Mixed Model-Analyses

| Fixed Factor | Weight | *SE* | *df* | *t* | *p* |
| --- | --- | --- | --- | --- | --- |
|  |  |  |  |  |  |
| *Base Model* |  |  |  |  |  |
| Intercept | 767.8 | 8.7 | 188.0 | 88.09 | < 0.001 |
| Prime (P) | -0.1 | 0.7 | 2352.9 | -0.19 | 0.849 |
| Target (T) | -4.4 | 1.2 | 190.2 | -3.79 | < 0.001 |
| P × T | -4.5 | 0.7 | 9375.6 | -6.63 | < 0.001 |
|  |  |  |  |  |  |
| *Step 1* |  |  |  |  |  |
| Intercept | 767.7 | 8.7 | 188.0 | 88.15 | < 0.001 |
| Prime (P) | -0.1 | 0.7 | 2673.1 | -0.13 | 0.899 |
| Target (T) | -4.1 | 0.6 | 40296.2 | -6.42 | < 0.001 |
| P × T | -3.5 | 0.6 | 40296.0 | -5.42 | < 0.001 |
| *Step 2* |  |  |  |  |  |
| Intercept | 767.1 | 8.7 | 187.9 | 87.78 | < 0.001 |
| Prime (P) | -0.2 | 0.7 | 3047.2 | -0.32 | 0.7475 |
| Target (T) | -3.6 | 0.7 | 40294.2 | -4.89 | < 0.001 |
| Know (K) | 1.4 | 0.8 | 9656.7 | 1.79 | 0.0732 |
| P × T | -3.4 | 0.7 | 40294.0 | -5.15 | < 0.001 |
| K × T | -1.2 | 0.7 | 40294.1 | -1.72 | 0.0853 |
| *Step 3* |  |  |  |  |  |
| *…* |  |  |  |  |  |
| P × K | -0.7 | 0.8 | 400.3 | -0.94 | 0.350 |
| P × T × K | -0.9 | 0.8 | 40274.6 | -1.17 | 0.244 |
|  |  |  |  |  |  |
| *Base Model (without unknown primes)* | | |  |  |  |
| Intercept | 768.5 | 8.7 | 188.0 | 88.55 | < 0.001 |
| Prime (P) | -0.6 | 0.8 | 5486.2 | -0.74 | 0.458 |
| Target (T) | -4.8 | 0.7 | 29203.4 | -6.48 | < 0.001 |
| P × T | -3.8 | 0.7 | 29203.2 | -5.09 | < 0.001 |

*Note*. Coding of predictor variables was as follows: *Prime* (P): -1 = hypothetical “old”, +1 = hypothetical “new”; *Target* (T): -1 = old, +1 = new; *Know* (K): -1 = “unknown”, +1 = “known or seen”. For further explanations, see text.

**References**

Bates, D., Maechler, M., Bolker, B., & Walker, S. (2015). Fitting linear mixed-effects models using lme4. *Journal of Statistical Software, 67*(1), 1-48. <https://doi.org/10.18637/jss.v067.i01>

Kuznetsova, A., Brockhoff, P. B., & Christensen, R. H. B. (2016). *lmerTest: Tests in linear mixed effects models. R package version 2.0-32.* <https://doi.org/https://CRAN.R-project.org/package=lmerTest>

R-Core-Team. (2016). *R: A language and environment for statistical computing. R Foundation for Statistical Computing, Vienna, Austria*
